# Long-term neuroendocrine consequences of early corticosteroid use in children

**DOI:** 10.1097/MS9.0000000000004344

**Published:** 2025-11-14

**Authors:** Mir Raza Ali, Iman Osman Abufatima

**Affiliations:** a Department of Medicine, Jinnah Postgraduate Medical Centre, Karachi, Pakistan; b Department of Medicine, University of Medical Sciences and Technology, Khartoum, Sudan

*Dear Editor,*

Early corticosteroid exposure, whether antenatal, neonatal, or prolonged postnatal therapy, can program the hypothalamic–pituitary–adrenal (HPA) axis and change cortisol control throughout infancy and childhood^[1,2]^. Several large systematic reviews show that antenatal corticosteroids reduce severe neurodevelopmental impairment in extremely preterm infants but increase the risk of neurocognitive or behavioral disorders when administered before late preterm or term birth, raising concerns about off-target HPA and brain programming effects^[1]^.

Persistent HPA hypoarousal is supported as a potential mechanism for subsequent neuroendocrine changes by prospective newborn studies that show lowered resting and post-stressor cortisol during the first year of life following antenatal corticosteroid exposure^[2]^.

Both the type and timing of glucocorticoids determine long-term risk, as evidenced by observational and systematic data on postnatal systemic steroids, especially dexamethasone, which link neonatal exposure to worse pulmonary and cognitive outcomes in childhood and adolescence^[3]^.

Glucocorticoid-induced adrenal insufficiency (GI-AI), growth effects, altered body composition, diminished bone mineral density, and metabolic shifts following high cumulative or chronic steroid administration are among the clinically significant endocrine sequelae reported in recent pediatric series.^[4,5]^ Somatic endocrine sequelae may outlast clinical therapy, as evidenced by a 2024 cross-sectional follow-up of children treated with high-dose systemic corticosteroids for childhood interstitial lung disease. The study found a slight increase in fat mass and altered bone mineral density in adulthood, as well as a temporary height standard deviation score reduction at treatment cessation^[5]^.

Narrative and evidence-synthesis reviews also point out that all steroid formulations and methods can suppress the HPA axis, and that recovery is varied, demanding careful tapering, baseline and stimulated cortisol monitoring, and adrenal crisis education^[4]^.

Beyond the suppression of HPA, a growing body of research on humans and animals links early exposure to glucocorticoids to altered neurodevelopmental trajectories through glucocorticoid receptor-mediated effects on hippocampus development, synaptic architecture, and stress-response circuitry alterations that may later show up as emotional, behavioral, or cognitive problems^[1–3]^. Crucially, the ratio of long-term risk to benefits varies depending on the indication and gestational age; prenatal steroids can still save the life of extremely premature infants, but their use should be avoided if it occurs carelessly or late in pregnancy^[1,6]^.

Practically speaking, clinicians should adopt low threshold follow-up for growth, pubertal timing, bone health, and stress-response evaluation in exposed children, record any perinatal or early childhood systemic steroid exposure in the child’s medical file, and advise families about potential long-term endocrine and neurodevelopmental problems^[4,5,7]^.

GI-AI risk can be decreased in situations when extended or high-dose medication is inevitable by transferring to shorter-acting preparations (such as hydrocortisone instead of longer-acting synthetic steroids) and arranging a slow weaning while monitoring adrenal function.^[4,6]^

In conclusion, current high-quality research and reviews support the concept that early corticosteroid exposure can have significant, often long-lasting neuroendocrine and somatic consequences, and that these concerns should guide prudent steroid use, thorough documentation, and systematic long-term follow-up. This manuscript complies with TITAN Guidelines, 2025, declaring no use of AI^[8]^.

## Data Availability

All data supporting the findings of this study are available within the article. The study is based on previously published literature; no new data were generated or analyzed.
